# Twins with different personalities: STAT5B—but not STAT5A—has a key role in BCR/ABL-induced leukemia

**DOI:** 10.1038/s41375-018-0369-5

**Published:** 2019-01-24

**Authors:** Sebastian Kollmann, Eva Grundschober, Barbara Maurer, Wolfgang Warsch, Reinhard Grausenburger, Leo Edlinger, Jani Huuhtanen, Sabine Lagger, Lothar Hennighausen, Peter Valent, Thomas Decker, Birgit Strobl, Mathias Mueller, Satu Mustjoki, Andrea Hoelbl-Kovacic, Veronika Sexl

**Affiliations:** 1 Institute of Pharmacology and Toxicology, University of Veterinary Medicine Vienna, 1210 Vienna, Austria; 2 Hematology Research Unit Helsinki, Department of Clinical Chemistry and Hematology, University of Helsinki and Helsinki University Hospital Comprehensive Cancer Center, P.O.Box 700, 00290 Helsinki, Finland; 3 Unit of Laboratory Animal Pathology, University of Veterinary Medicine Vienna, 1210 Vienna, Austria; 4 Laboratory of Genetics and Physiology, National Institute of Diabetes and Digestive and Kidney Diseases, National Institutes of Health, Bethesda, MD 20892 USA; 5 Department of Internal Medicine I, Division of Hematology and Hemostaseology, Comprehensive Cancer Center, Medical University of Vienna, 1090 Vienna, Austria; 6 Ludwig Boltzmann Cluster Oncology, Medical University of Vienna, 1090 Vienna, Austria; 7 Max F. Perutz Laboratories (MFPL), University of Vienna, 1030 Vienna, Austria; 8 Department for Biomedical Sciences Institute of Animal Breeding and Genetics, University of Veterinary Medicine Vienna, 1210 Vienna, Austria

**Keywords:** Acute lymphocytic leukaemia, Cell signalling

## Abstract

Deregulation of the Janus kinase/signal transducers and activators of transcription (JAK/STAT) signaling pathway is found in cancer with STAT5A/B controlling leukemic cell survival and disease progression. As mutations in *STAT5B*, but not *STAT5A*, have been frequently described in hematopoietic tumors, we used BCR/ABL as model systems to investigate the contribution of STAT5A or STAT5B for leukemogenesis. The absence of STAT5A decreased cell survival and colony formation. Even more drastic effects were observed in the absence of STAT5B. STAT5B-deficient cells formed BCR/ABL^+^ colonies or stable cell lines at low frequency. The rarely evolving *Stat5b*^*−/−*^ cell lines expressed enhanced levels of BCR/ABL oncoprotein compared to wild-type cells. In line, *Stat5b*^*−/−*^ leukemic cells induced leukemia with a significantly prolonged disease onset, whereas *Stat5a*^*−/−*^ cells rapidly caused a fatal disease superimposable to wild-type cells. RNA-sequencing (RNA-seq) profiling revealed a marked enhancement of interferon (IFN)-α and IFN-γ signatures in *Stat5b*^*−/−*^ cells. Inhibition of IFN responses rescued BCR/ABL^+^ colony formation of *Stat5b*^*−/−*^-deficient cells. A downregulated IFN response was also observed in patients suffering from leukemia carrying *STAT5B* mutations. Our data define STAT5B as major STAT5 isoform driving BCR/ABL^+^ leukemia. STAT5B enables transformation by suppressing IFN-α/γ, thereby facilitating leukemogenesis. Our findings might help explain the high frequency of *STAT5B* mutations in hematopoietic tumors.

## Introduction

Janus kinase/signal transducers and activators of transcription (JAK/STAT) signaling has been implicated in multiple forms of solid as well as hematologic cancers [1]. In particular, the constitutive activation of JAK/STAT signaling is found in several forms of leukemia [2–7] (http://cancergenome.nih.gov/). We have identified the transcription factors STAT5A/B as critical nodes in the signaling network downstream of the leukemia-associated BCR/ABL oncogene [8, 9]. The STAT5 locus is comprised of two genes, *STAT5A* and *STAT5B*. So far, only little is known of STAT5A- or STAT5B-specific functions in hematopoietic cells. In non-hematopoietic cells, STAT5A and STAT5B fulfill non-redundant functions. STAT5A, but not STAT5B, is essential for mammary gland development and prolactin signaling [10, 11]. On the other hand, STAT5B mediates growth hormone signaling and *Stat5b*-knockout mice show reduced body growth [12, 13]. In BCR/ABL-driven lymphoid as well as myeloid leukemia, the combined loss of STAT5A and STAT5B halts disease, the requirement for STAT5A/B extending to the leukemic stem cell compartment [9].

In leukemia, STAT5A and STAT5B may have individual functions as the constitutive activation of either protein is capable to inflict a disease of a specific phenotype. In mice, the expression of a constitutively active (ca) version of STAT5A (caSTAT5A) induces an aggressive multi-lineage leukemia [14]. The expression of caSTAT5B or the overexpression of wild-type (wt) STAT5B selectively disturbs lymphopoiesis resulting in profound expansion of B and T cell numbers [15–17]. In NPM-ALK-driven neoplasms, silencing of STAT5B blocks and silencing of STAT5A supports the disease [18, 19]. So far it remains unclear how STAT5A and STAT5B exert their independent functions despite sharing 94% structural homology. Complete *Stat5a* and *Stat5b* single knockout mice were generated which offer the possibility to dissect individual functions of these proteins [10, 13].

Genome-wide screening of mutations in cancers revealed that mutations affect *STAT5B* at a much higher frequency than *STAT5A* (https://cancer.sanger.ac.uk/cosmic). These mutations are confined to hematological disorders (mainly T cell and natural killer T cell leukemias and lymphomas).

In 2013, Rajala et al. [20] identified a *STAT5B* missense mutation (encoding a STAT5B^N642H^ mutant) in cases of large granulocytic lymphocytic (LGL) leukemia. The same mutation was later on also discovered in acute T cell leukemia [21, 22], T-prolymphocytic leukemia [23], and hepatosplenic T cell lymphoma [24]. By now, according to the COSMIC database, somatic STAT5B^N642H^ mutation was identified in 11 types of leukemia currently summing up to prevalence in more than 90 patients, the incidence rising (cancer.sanger.ac.uk/cosmic/).

The STAT5B^N642H^ mutation affects the Src homology 2 domain and reportedly increases the stability of the STAT5B dimer [25]. As a result, the transcriptional activity of STAT5B is markedly increased [21]. In line, the presence of a STAT5B^N642H^ mutant in BA/F3 cells confers interleukin-3-independent growth [26, 27]. Only recently, a STAT5B^N642H^ transgenic mouse model was generated recapitulating the T cell neoplasia phenotype observed in human patients [27]. These observations indicate a yet underestimated role of STAT5B in human and murine leukemogenesis.

Here we investigated why mutations in human cancers are predominantly found in *STAT5B* and not in *STAT5A*. We used knockout mice, which either lack STAT5A or STAT5B [10, 13]. Using a combination of transformation studies in vitro and in vivo, we identify STAT5B as the dominant isoform allowing leukemic cell transformation and propagation of disease. Transcriptional profiling revealed that IFN-α and IFN-γ pathways are pronouncedly upregulated in *Stat5a*^*−/−*^ and *Stat5b*^*−/−*^ cells, the extent being higher in *Stat5b*^*−/−*^ cells. Blockage of IFN-α and IFN-γ signaling restored their capability to transform. In line, transcriptional analysis of *STAT5B*-mutated leukemia patient samples revealed a downregulated IFN response. Taken together, our data point at a dominant role of STAT5B in suppressing IFN-α and IFN-γ signaling during leukemic transformation.

## Material and methods

### Primary CML patient samples

Primary leukemic cells were obtained from patients with chronic myeloid leukemia (CML) at routine blood and bone marrow (BM) examinations after informed consent was given in compliance with the Declaration of Helsinki. Peripheral blood and BM mononuclear cells were isolated using Ficoll. Samples were analyzed for BCR/ABL mutations and BCR/ABL messenger RNA (mRNA) levels according to the international scale as reported [28]. Use of human samples was approved by the ethics committee of the Medical University of Vienna (ethics committee no. 1184/2014) and is in compliance with Austrian legislation.

### Primary T-LGLL patient samples

The study was undertaken in compliance with the principles of the Declaration of Helsinki and was approved by the ethics committees in the Helsinki University Central Hospital (Helsinki, Finland). All patients and healthy controls gave written informed consents. All patients met the criteria of T-cell large granular lymphocytic leukemia (T-LGLL) as defined by the World Health Organization (2008). STAT5B mutations were identified with exome sequencing and validated by capillary sequencing, as previously described [20, 29, 30]. Patient samples harboring the following mutations were included in this study: STAT5B^Y665F^, STAT5B^Q706L^, and STAT5B^S715F^.

### Mice

*Stat5a*^*−/−*^ [10], *Stat5b*^*−/−*^ [13], NSG (NOD.Cg-*Prkdc*^*scid*^ *Il2rg*tm ^*1Wjl*^/SzJ; license BMWF 68.205/0093-WF/V/3b/2015), and NOG-F mice (NOD.Cg*-Prkdcscid Il2rgtm1Sug/*JicTac, Taconic Biosciences A/S) were maintained under SPF conditions at the University of Veterinary Medicine Vienna. All animal experiments were conducted in 6- to 9-week-old mice and approved by institutional ethics and animal welfare committee and the national authority according to §§26ff. of Animal Experiments Act, Tierversuchsgesetz 2012 – TVG 2012 (license BMWF-68.205/0218-II/3b/2012).

### Transplantation of leukemic cells and new-born infections

#### Subcutaneous injection

NSG mice were injected into the flanks with 1 × 10^6^ BCR/ABL^p185+^ cells. After 8–12 days, tumor nodules were palpable and tumor sizes were measured every other day with a slide caliper and calculated using the formula: *a* × *b*/2 (*a*: length of tumor; *b*: tumor width). Tumor weights were determined after sacrificing the mice.

#### Intravenous injection

BCR/ABL^p185+^ cells (1 × 10^4^) were injected intravenously (i.v.) into NSG mice. Sick mice were sacrificed upon paralysis of hind legs and analyzed for spleen weights, white blood cell counts, and the presence of leukemic cells in BM, spleen, and blood.

BCR/ABL^p185+^ cells (5 × 10^4^) were injected i.v. into NOG-F mice. Mice were sacrificed at day 5, day 8, and day 11 upon injection and analyzed for the presence of leukemic cells in BM, spleen, and blood.

#### New-born infections

Ab-MulV was subcutaneously injected into new-born mice (24–48 h after birth), conducted as described [31].

### RNA-seq analysis

#### Murine data

For RNA-sequencing (RNA-seq) of immortalized *Stat5a*^*−/−*^, *Stat5b*^*−/−*^, and wt BCR/ABL^p185+^ cell lines, libraries from mRNA of these cell lines (*n* = 1 per genotype in three technical replicates) were prepared using the Lexogen SENSE mRNA-Seq library preparation kit (Lexogen, Vienna, Austria). Single-end, 50 bp sequencing was performed on an Illumina HiSeq-2500 sequencer (Illumina, San Diego, CA, USA). After quality control of raw data with FastQC and removement of adapters and low-quality reads with Trimmomatic (version 0.36), reads were mapped to the GENECODE M13 genome using STAR (version 2.5.2b) with default parameters. Counts for union gene models were obtained using featureCounts from the Subread package (version 1.5.1). Differentially expressed (Benjamin–Hochbert corrected *p* value (*p*-adjust) < 0.05 and fold change >2) genes were identified using DESeq2 (version 1.18.1). Genset enrichment analysis (GSEA) against Molecular Signature Database (MSigDB) hallmark gene sets with log 2 fold-change ranked lists from differential expression analysis (DEA) was utilized to determine significantly deregulated pathways (absolute normalized enrichment score (NES) >1, false discovery rate (FDR) <0.25). To demonstrate stronger increase of the type I and type II IFN response pathway in *Stat5b*^*−/−*^ than in *Stat5a*^*−/−*^ BCR/ABL^p185+^ cell lines, log 2 fold changes and adjusted *p* values of the DEA (*Stat5a*^*−/−*^ vs. wt and *Stat5b*^*−/−*^ vs. wt) of genes that contribute to core enrichment in either of the two GSEA analysis are shown.

The RNA-seq data reported in this article have been deposited in the Gene Expression Omnibus database (Accession ID: GSE121246).

#### Human patient data

For RNA-seq of STAT5B mutant (1 CD4^+^, 1 CD4^+^CD8^+^, and 2 CD8^+^) and wt (13 CD8^+^) T-LGLL samples were prepared using miRNeasy mini kit (Qiagen) and Nucleospin RNA II kit (Macherey-Nagel). Sequencing libraries were sequenced using paired-end 100 bp read format on an Illumina HiSeq 2000 instrument (Illumina). Paired-end reads passing the pre-processing were aligned to human reference genome build 38 (EnsEMBL v82) using STAR (version 2.5.2b) with the default two-pass per-sample mapping settings. Reads were then sorted by coordinate using the SortSAM and PCR duplicates were marked using the MarkDuplicate module of the Picard toolkit. Mapped reads were assigned to gene features (EnsEMBL v82) using FeatureCounts by allowing multi-mapping reads and assignment of a read to more than one overlapping feature.

Differentially expressed (*p*-adjust <0.05 and fold change >2) genes were identified with edgeR (version 3.22.3) with common dispersion with default parameters. GSEA analysis was done as described above.

### Colony formation assay with or without blocking of IFN-α/β and IFN-γ responses and growth curve

BM cells were infected with Ab-MulV, BCR/ABL^p185^, or BCR/ABL^p210^ virus containing supernatant as previously described [8]. Cells (1 × 10^6^) were seeded in methylcellulose without supplement of cytokines (MethoCult, 03231, STEMCELL Technologies, Vancouver, BC, Canada). For IFN signaling blocking experiments, 10 µg/ml immunoglobulin G (IgG) control antibody (02-6102, Invitrogen, San Diego, CA, USA) or 10 µg/ml IFNAR1-blocking antibody (eBioscience, San Diego, CA, USA) or 10 µg/ml IFN-γ-blocking antibody (Becton-Dickinson, Franklin Lakes, NJ, USA) or a combination of both (in total 10 µg/ml) was directly added to the methylcellulose (MethoCult, 03231, STEMCELL Technologies). Colonies were counted after 9–14 days and photographed using a ChemiDoc^TM^ Touch Imaging System (Bio-Rad, Hercules, CA, USA). Representative colonies were picked for fluorescence-activated cell sorting (FACS) analysis.

For growth analysis, 5 × 10^5^ BCR/ABL^p185+^ cells were seeded and 100 µl of cell suspension was used for counting via flow cytometry at indicated time points. Cells were split every third day and filled up with fresh medium to maintain exponential growth.

Competitive growth analysis of short hairpin RNA (shRNA)-mediated STAT5A and STAT5B knockdowns was performed in K562 cells. Percentages of green fluorescent protein-positive (GFP^+^) cells (starting point/starting value in all samples ~80% GFP^+^) were analyzed at the indicated time points via flow cytometry. All time points were normalized to the percentage of GFP^+^ cells at day 5 of the respective sample. Cells were split every third day and filled up with fresh medium to maintain exponential growth.

### Statistical analyses

Kruskal–Wallis test (followed by Dunn’s test), one-way analysis of variance (followed by Tukey's multiple comparison test), log-rank (Mantel–Cox) test, Wilcoxon–Mann–Whitney test, *χ*^2^ test and assessment of half maximal inhibitory concentration (IC_50_) values were performed using GraphPad Prism^®^ Software version 5.04 and 6.02. Statistical significance is indicated for each experiment specifically (*p < 0.05; **p < 0.01; ***p < 0.001).

## Results

### Overexpression of *Stat5b* enhances cell proliferation of BCR/ABL^+^ cells

**Fig. 1:**
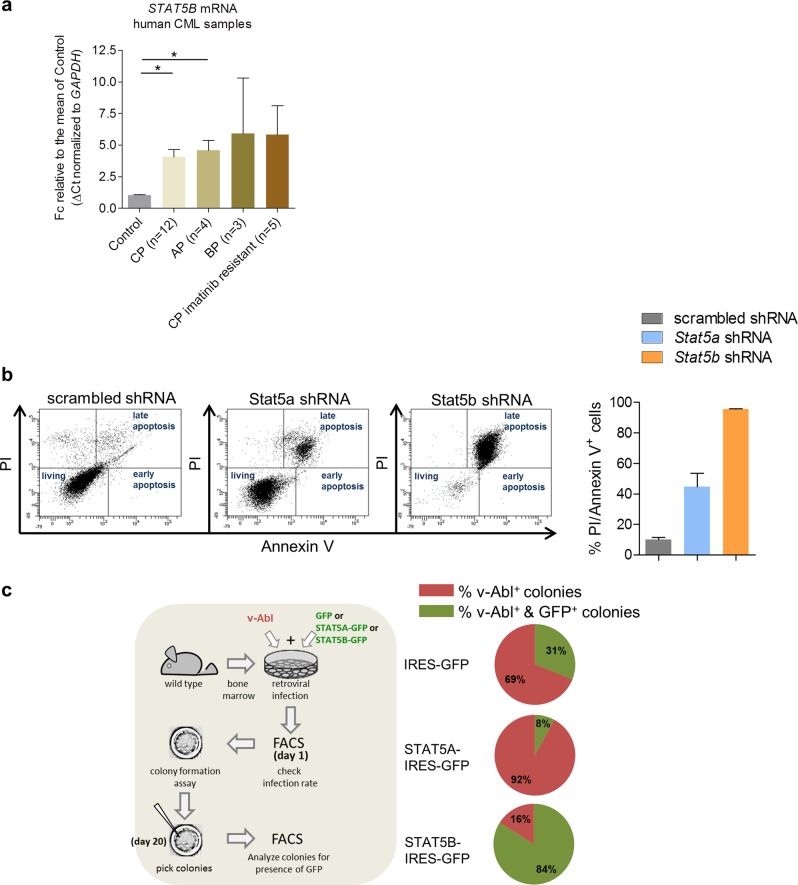
STAT5B controls transformation and survival of BCR/ABL^+^ cells. **a** qPCR for *STAT5B* in human CML samples. Fold change of *STAT5B* mRNA levels compared to the mean level of control bone marrow (BM) samples. Control BM was derived from healthy individuals (*n* = 3); untreated CML patients in chronic phase (CP) (*n* = 12), acute phase (AP) (*n* = 4) or blast phase (BP) (*n* = 3) and relapsed imatinib-resistant patients in CP (*n* = 5). Error bars represent mean ± SEM. Results were normalized to *GAPDH* mRNA expression. Levels of significance were calculated using Kruskal–-Wallis test followed by Dunn’s test. **b** Representative FACS plots (Annexin-V/PI stainings) of BCR/ABL^p185+^ cells with shRNA-mediated knockdown of *Stat5a* or *Stat5b*. Scrambled shRNA was used as control. Right: Percentages of Annexin-V/PI–double positive BCR/ABL^p185+^ cell lines with shRNA-mediated knockdown of *Stat5a* or *Stat5b* (*n* = 2 cell lines/construct). Error bars represent mean ± SEM. Levels of significance were calculated using Kruskal–Wallis test followed by Dunn’s test. **c** Co-infection of BM cells with Ab-MuLV (encoding v-Abl) and vectors expressing STAT5A or STAT5B (infection rates are provided in Supplementary Figure 1c) were plated in growth factor-free methylcellulose (*n* = 2 cell lines/construct). Mean percentages of colonies carrying STAT5A and STAT5B are indicated in pie charts. STAT signal transducers and activators of transcription, BM bone marrow, wt wild type, qPCR quantitative PCR, CML chronic myeloid leukemia, mRNA messenger RNA, PI propidium iodide, shRNA short hairpin RNA

We have shown that the levels of STAT5A increase during progression of CML [32]. Similarly, the expression of STAT5B increases significantly in samples derived from CML patients when they reach the accelerated phase (AP) or chronic phase (CP). We observe a tendency of STAT5B upregulation in samples derived from patients in blast crisis and in those who became imatinib-resistant during CP (Fig. 1a). To test whether STAT5A or STAT5B control survival of BCR/ABL^+^ leukemic cells, we expressed *Stat5a*, *Stat5b*, or the empty vector (henceforth called “GFP” for simplicity) in *v-Abl*-transformed cells. Enforced expression of STAT5A or STAT5B provided a proliferative advantage, the effect of STAT5B overexpression being more pronounced (Supplementary Figure 1a). Loss-of-function studies in murine BCR/ABL^p185+^ cells complemented these experiments; knockdown of *Stat5b* induced apoptosis, whereas the effects of the *Stat5a*-directed shRNA were less pronounced shortly upon sorting of infected cells (Fig. 1b). To understand the effect of STAT5A and STAT5B on the initial transformation process, we infected BM cells with a retrovirus encoding for *v-Abl* in combination with a retrovirus conferring either *Stat5a* or *Stat5b* expression linked via internal ribosome entry site to GFP. Mean levels of expression per cell of either vector were superimposable (Supplementary Figure 1b). Again, the empty GFP vector served as control. Against our expectations, the concomitant expression of *Stat5a* and the *v-Abl* oncogene repressed colony formation compared to the empty vector setting (Fig. 1c). In contrast, the frequency of colonies expressing both v-Abl and STAT5B was increased.

### *Stat5b* promotes initial transformation and cell proliferation

**Fig. 2:**
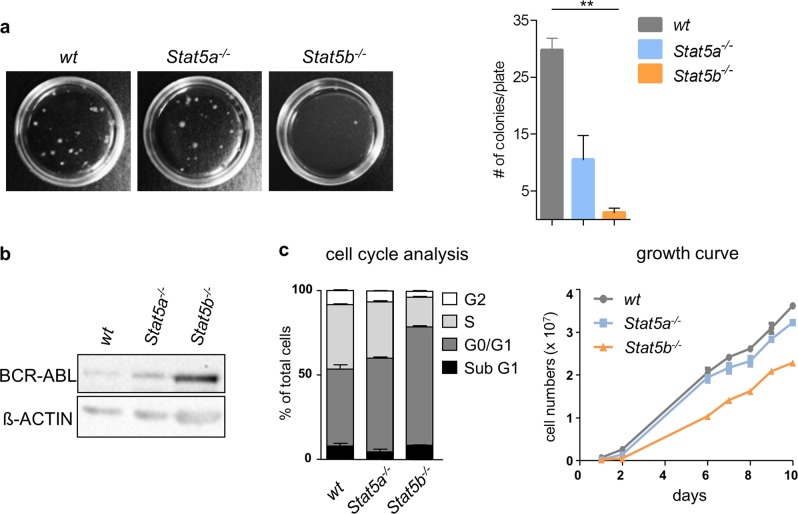
STAT5B controls initial transformation and cell proliferation. **a** BM cells of wt, *Stat5a*^*−/−*^, or *Stat5b*^*−/−*^ mice were infected with a retrovirus encoding BCR/ABL^p185^ and plated in growth factor-free methylcellulose. Representative pictures of colony formation assays (CFA) are depicted. Bar graph summarizes data obtained from 4 cell lines/genotype each in technical duplicates. Error bars represent mean ± SEM. Levels of significance were calculated using Kruskal–Wallis test followed by Dunn’s test. **b** Protein levels of BCR/ABL in wt, *Stat5a*^*−/−*^, or *Stat5b*^*−/−*^ cell lines as determined by immunoblotting. One representative blot is shown. β-Actin served as a loading control. **c** Cell cycle profiles analyzed by PI-staining of BCR/ABL^p185^-transduced cell lines (*n* = 2 cell lines/genotype). Error bars represent mean ± SEM. Right: Growth curves of established wt, *Stat5a*^*−/−*^, or *Stat5b*^*−/−*^ BCR/ABL^p185+^ cell lines (*n* = 2 cell lines/genotype). Error bars represent mean ± SEM. Levels of significance were calculated using Kruskal–Wallis test followed by Dunn’s test. STAT signal transducers and activators of transcription, BM bone marrow, wt wild type, PI propidium iodide

To substantiate the findings, we next employed mouse models with specific deletions for either STAT5A or STAT5B [10, 13]. BM cells were infected with a retrovirus encoding BCR/ABL^p185^ and plated in growth factor-free methylcellulose. Whereas the absence of *Stat5a* reduced colony numbers about two-thirds, hardly any colonies were derived from *Stat5b*-deficient BM (Fig. 2a). These differences were reflected by the frequency in the outgrowth of stable cell lines; in the absence of STAT5A, we received 10/17 stable (59%) cell lines, while only 4/14 (29%) stable cell lines were derived from STAT5B-deficient BM (*χ*^2^: 94.75, df: 4, *p* < 0.0001). Moreover, *Stat5b*-deficient cell lines grew out with a delay of ~12 weeks post infection, whereas *Stat5a*-deficient cells required about 6 weeks to form a stable cell line similar to wt lines, which succeeded to 100% to grow into stable cell lines (12/12). The absence of *Stat5a* and *Stat5b* required an enhanced BCR/ABL expression level (indicated by higher mean fluorescence intensity in the FACS analysis), which was also evident in western blot analysis (Supplementary Figure 2a and b). In line, the IC_50_ level for the BCR/ABL-inhibitor, imatinib were enhanced in *Stat5b*-deficient cells (Supplementary Figure 2b). When analyzing cell cycle progression of established cell lines by FACS, we observed consistently reduced numbers of cells in the S phase in *Stat5b*^*−/−*^ cells (Fig. 2c, left panel). Growth curves over a period of 10 days confirmed the reduced cell proliferation upon STAT5B deficiency (Fig. 2c, right panel). These data indicate a role for STAT5B during the initial transformation process and for cell proliferation driven by BCR/ABL.

### STAT5B is required for leukemogenesis and tumor formation in vivo

**Fig. 3:**
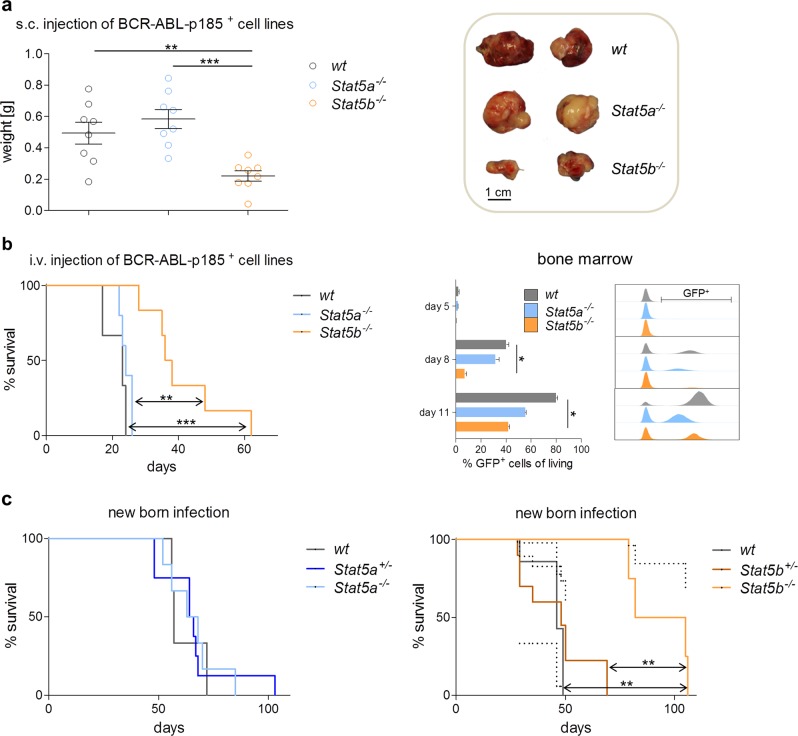
BCR/ABL-induced tumor formation in vivo is impaired upon loss of STAT5B. **a** Left: Subcutaneous injection of BCR/ABL^p185+^ established cell lines into NSG mice (*n* = 8 per genotype). Bar graph summarizes tumor weights. Levels of significance were calculated using one-way ANOVA. Error bars represent mean ± SEM. Right: Representative pictures of tumor sizes are depicted. **b** Kaplan–Meier plot of NSG mice that have received an intravenous injection (*i.v*.) of wt, *Stat5a*^*−/−*^, or *Stat5b*^*−/−*^ BCR/ABL^p185+^ cells (*n* ≥ 6 per genotype). Log-rank (Mantel–Cox) test was used to calculate levels of significance. Right: *I.v.* injection of wt, *Stat5a*^*−/−*^, or *Stat5b*^*−/−*^ BCR/ABL^p185+^ cells into NSG mice. Three mice per genotype were sacrificed on day 5, day 8, and day 11 upon injection of BCR/ABL^p185+^ cells. Quantitative analysis via FACS for GFP^+^ BCR/ABL^p185+^ cells in the BM (*n* = 3 per genotype and day) as well as representative histograms of each day and phenotype are provided. Error bars represent mean ± SEM. Levels of significance were calculated using Kruskal–Wallis test followed by Dunn’s test. **c** Survival plots after new-born infections using Ab-MulV (encoding v-abl) comparing left: wt, *Stat5a*^*+/−*^, and *Stat5a*^*−/−*^ mice (*n* ≥ 3 per genotype) or right: wt, *Stat5b*^*+/−*^, and *Stat5b*^*−/−*^ mice (*n* ≥ 4 per genotype). Log-rank (Mantel–Cox) test was used to calculate levels of significance. NSG NOD.Cg-*Prkdc*^*scid*^ *Il2rg*tm ^*1Wjl*^/SzJ, ANOVA analysis of variance, IFN interferon, STAT signal transducers and activators of transcription, wt wild type, GFP green fluorescent protein

Deletion of *Stat5a/b* impairs leukemic cell growth in vivo [8, 9, 33, 34]. To dissect the individual contribution of STAT5A and STAT5B to lymphoid tumor formation, we injected *Stat5a*^*−/−*^, *Stat5b*^*−/−*^, or wt BCR/ABL^p185+^ cell lines subcutaneously into NSG mice. *Stat5b*^*−/−*^ cells elicited significantly smaller tumors than *Stat5a*^*−/−*^ or wt cell lines (Fig. 3a). To model a systemic disease, we injected the cell lines intravenously into NSG mice. We observed a significantly prolonged survival of mice that had received *Stat5b*^*−/−*^ cells compared to the cohorts that were transplanted with *Stat5a*^*−/−*^ or wt cell lines (Fig. 3b, left panel). The later time point of disease onset in *Stat5b*^*−/−*^ transplanted mice was paralleled by reduced numbers of leukemic (CD19^+^) cells in the BM (Supplementary Figure 3a). Frequencies of leukemic cells in the blood and spleen as well as white blood cell counts (WBCs) were comparable to those of the other cohorts (Supplementary Figure 3a and b). To gain more insight into the kinetics of disease development, we repeated the experiment and followed expansion of GFP^+^ cells over time (Fig. 3b, right panel and Supplementary Figure 3c). Whereas leukemic cells were hardly present on day 5 in all experimental groups, a clear reduction of GFP^+^ leukemic cells was evident on day 8 in the BM, spleen, and in the peripheral blood in mice that had received *Stat5b*^*−/−*^ cells. In BM and blood, this difference extended to day 11.

To mimic disease development in people, we injected a retrovirus encoding *v-abl* intraperitoneally into new-born mice inducing a slowly evolving mono- or oligo-clonal disease. *Stat5a*^*−/−*^ and *Stat5b*^*−/−*^ mice and their heterozygous and wt littermates were injected 24 to 36 h after birth. *Stat5a*^*−/−*^, *Stat5a*^*+/−*^, and wt mice showed overlapping kinetics in the incidence of terminal disease. In contrast, *Stat5b*-deficient mice diseased significantly later than their *Stat5b*^*+/−*^ and wt littermates (Fig. 3c). These data verify the privileged role for STAT5B in BCR/ABL-induced leukemia in vivo.

### STAT5B suppresses IFN responses in BCR/ABL^+^ cells

To gain insights into how STAT5B interferes with BCR/ABL-induced leukemogenesis, we performed RNA-seq analysis. 881 genes were upregulated and 608 genes were downregulated exclusively in *Stat5b*^*−/−*^ cells, compared to wt cells (Supplementary Figure 4, Supplementary Tables 1 and 2).

**Fig. 4:**
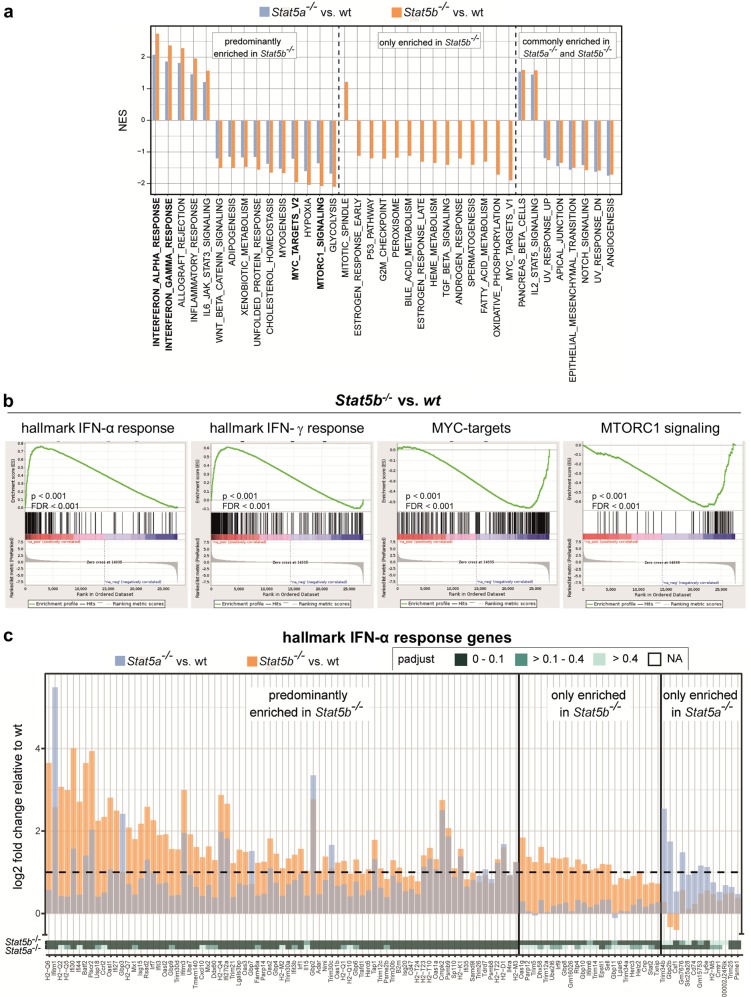
STAT5B suppresses interferon responses in BCR/ABL^+^ cells. RNA-seq analysis was performed on BCR/ABL^p185+^ cell lines derived from wt, *Stat5a*^*−/−*^ and *Stat5b*^*−/−*^ mice. **a** Significantly (NES >1, FDR <0.25) enriched hallmark gene sets obtained from GSEA of log 2 fold-change ranked gene lists from differential expression analysis of *Stat5b*^*−/−*^ (vs. wt) and *Stat5a*^*−/−*^ (vs. wt) BCR/ABL^p185+^ cell lines. Gene sets more enriched in *Stat5b*^*−/−*^ (left panel), only enriched in *Stat5b*^*−/−*^ (middle panel), and equally enriched in *Stat5b*^*−/−*^ and *Stat5a*^*−/−*^ (right panel) are shown. **b** Enrichment plots of the hallmark IFN-α response gene set from GSEA above and additional GSEA of differentially regulated genes between *Stat5b*^*−/−*^ and *Stat5a*^*−/−*^ BCR/ABL^p185+^ cell lines. **c** The change in expression levels of genes that contribute to core enrichment of the hallmark IFN-α response gene set in *Stat5b*^*−/−*^ (orange) and *Stat5a*^*−/−*^ (blue) BCR/ABL^p185+^ cell lines relative to wt BCR/ABL^p185+^ cell lines and the significance of this change (*p*-adjust, see legend) is shown. IFN interferon, STAT signal transducers and activators of transcription, wt wild type, NES normalized enrichment score, GSEA gene set enrichment analysis, *p*-adjust Benjamin–Hochbert corrected *p* value, FDR false discovery rate

GSEA revealed three distinct groups: we found pathways that are (i) significantly higher enriched in *Stat5b*^*−/−*^ than in *Stat5a*^*−/−*^ cells, (ii) those which are exclusively enriched in *Stat5b*^*−/−*^ cells and (iii) those which are commonly regulated in both genotypes (Fig. 4a). IFN signaling comprised a major deregulated pathway; IFN-α and IFN-γ responses were upregulated in *Stat5a*^*−/−*^ and *Stat5b*^*−/−*^ cells, the extent being significantly higher in *Stat5b*^*−/−*^ cells (Fig. 4a and Supplementary Figure 5). Downregulated pathways contained MYC-, MTORC1 signaling, and glycolysis—in line with the proposed role of STAT5 in tumor formation (Fig. 4a). Enrichment plots underline the increased prevalence of hallmark IFN-α and IFN-γ genes and the decreased abundance of MYC targets and MTORC1 signaling in *Stat5b*^*−/−*^ (Fig. 4b) and of MTORC1 targets in *Stat5a*^*−/−*^ cells (Supplementary Figure 6a and b).

**Fig. 5:**
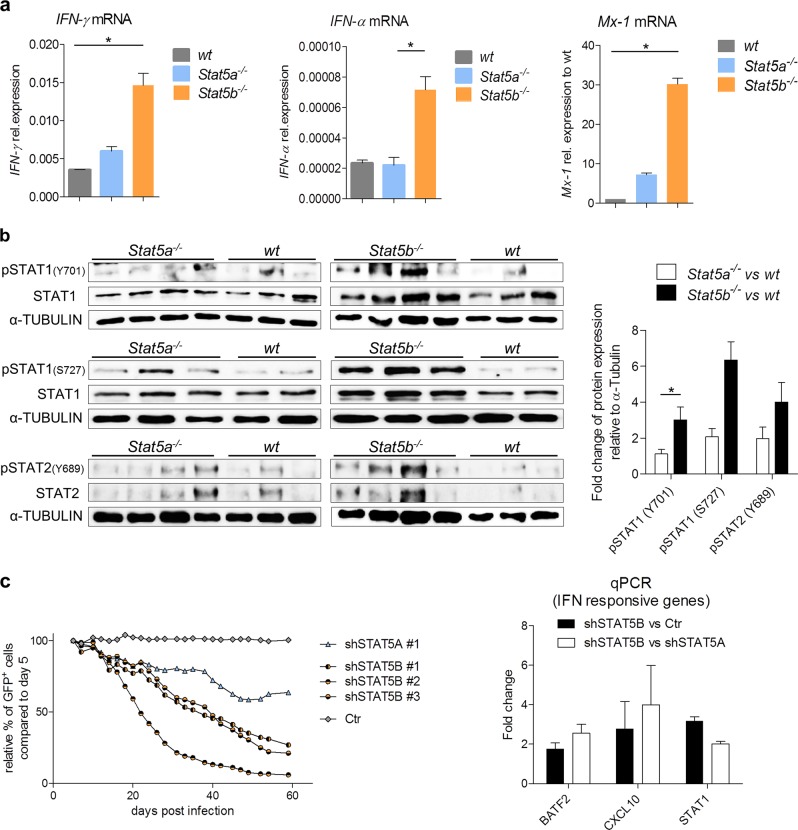
IFN-α and IFN-γ responses are impaired in BCR/ABL^p185+^ *Stat5b*^*−/−*^ cells. qPCRs of wt, *Stat5a*^*−/−*^, or *Stat5b*^*−/−*^ BCR/ABL^p185+^ cell lines for **a** *IFN-*γ, *IFN-α*, and *Mx-1* mRNA expression (*n* ≥ 3 cell lines/genotype). Error bars represent mean ± SEM. Levels of significance were calculated using Kruskal–Wallis test followed by Dunn’s test. **b** Protein levels of pSTAT1(pS727, pY701), pSTAT2(pY698), STAT1 and STAT2 in wt, *Stat5a*^*−/−*^, or *Stat5b*^*−/−*^ BCR/ABL ^p185+^ cells as determined by immunoblotting (each lane represents an independent cell line). Right*:* Densitometric analysis of pSTAT1(pS727, pY701) and pSTAT2(pY698) protein levels normalized to total protein loaded and wt. Error bars represent mean ± SEM. Levels of significance were calculated via Wilcoxon–Mann–Whitney test. **c** Outgrowth of K562 cells expressing shRNAs against STAT5A (*n* = 1), STAT5B (*n* = 3), and Control (Ctr = Renilla, *n* = 1), in the presence of their non-transfected counterparts. Percentages of shRNA-expressing cells were detected by GFP expression and analyzed by flow cytometry over a period of 2 months post infection. Right: K562 cells were treated with shSTAT5A, shSTAT5B, or Ctr shRNA and sorted for GFP^+^ cells. Fold change of mRNA expression levels were detected via qPCR for *BATF2*, *CXCL10*, and *STAT1*. Error bars represent mean ± SEM. Levels of significance were calculated via Wilcoxon–Mann–Whitney test. IFN interferon, qPCR quantitative PCR, BM bone marrow, STAT signal transducers and activators of transcription, wt wild type, shRNA short hairpin RNA, mRNA messenger RNA, GFP green fluorescent protein

Genes that contribute to the core enrichment of hallmark IFN-α and IFN-γ response were subdivided into those which are predominantly enriched in *Stat5b*^*−/−*^, those which are only enriched in *Stat5b*^*−/−*^, and those which are only enriched in *Stat5a*^*−/−*^ cells. Key IFN-α- and IFN-γ-responsive genes were found in the “only enriched in *Stat5b*^*−/−*^” group (such as *Stat2* or *Irf9*) and in the “predominantly enriched in *Stat5b*^*−/*^” group (such as *Mx-1*, *Mx-2*, or *Irf7*) (Fig. 4c and Supplementary Figure 7). The significantly higher impact on IFN-dependent gene regulation upon loss of STAT5B was verified by quantitative PCR (qPCR) for *IFN-γ* and *IFN-α* as well as the IFN-α-dependent immediate gene *Mx-1* (Fig. 5a). STAT1 and STAT2 are major transcriptional regulators downstream of IFN signaling. Cells lacking STAT5B display enhanced pSTAT1 and pSTAT2 levels compared to wt or *Stat5a*^*−/−*^ cell lines (Fig. 5b). To test whether the effect extends to human BCR/ABL^+^ leukemia, we ablated STAT5A or STAT5B via shRNA-mediated knockdowns in K562 cells. Efficiencies of knockdowns were verified by immunoblotting (Supplementary Figure 8a). We monitored the outgrowth of shRNA-expressing cells in the presence of their non-transfected counterparts. Whereas loss of STAT5A led to a mild effect, the loss of STAT5B associated with a significant growth disadvantage (Fig. 5c, left panel and Supplementary Figure 8b). In line with the data obtained in the murine system, the expression levels of key interferon hallmark genes were markedly upregulated in STAT5B-deficient K562 cells (Fig. 5c, right panel). Taken together, these data point at a suppressive role of STAT5B in IFN-α and IFN-γ pathways upon transformation.

### Blocking of type I or type II IFN signaling enables *Stat5b*^*−/−*^ cells to form colonies

**Fig. 6:**
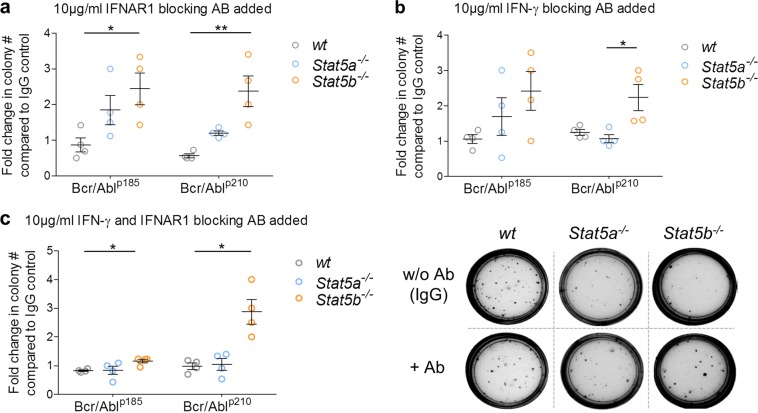
Blockage of IFN-α or IFN-γ signaling restores the capacity of *Stat5b*^*−/−*^ cells to form colonies. BM cells of wt, *Stat5a*^*−/−*^ or *Stat5b*^*−/−*^ mice were infected with a retrovirus encoding BCR/ABL^p185^ or BCR/ABL^p210^ and plated in growth factor-free methylcellulose. In addition 10 µg/ml antibodies (Ab) blocking **a** IFNAR1 or **b** IFN-γ or **c** combination of both (in total 10 µg/ml) were added (*n* = 4 per genotype). Levels of significance were calculated using Kruskal–Wallis test followed by Dunn’s test. Error bars represent means ± SEM. Right: representative pictures of CFA. IFN interferon, BM bone marrow, STAT signal transducers and activators of transcription, wt wild type, GSEA geneset enrichment analysis, CFA colony formation assays

To investigate the functional consequences of the STAT5–IFN axis, we next asked whether the elevated IFN signaling in *Stat5b*^*−/−*^ cells accounts for the reduced colony formation upon transformation by BCR/ABL^p185^ or BCR/ABL^p210^ oncogenes. To do so, we performed colony formation assays in the presence of IFNAR1 and/or IFN-γ antibodies. Treatment with anti-IFNAR1 increased the number of colonies compared to IgG controls in *Stat5b*^*−/−*^ cells (Fig. 6a). In line with the RNA-seq data, the effects were less pronounced in *Stat5a*^*−/−*^ cells. Blocking of IFN-γ elicited a comparable picture (Fig. 6b) as did the simultaneous blocking of type I and type II IFN signaling (Fig. 6c; *p* = 0.0208). These data suggest that blockage of IFNAR1 signaling or IFN-γ restores the transformation capacity of *Stat5b*^*−/−*^ cells.

### IFN responses are downregulated in leukemic patients harboring *STAT5B* mutations

Genome-wide screening of cancer patients is identifying increasing numbers of patients harboring somatic mutations in *STAT5B* (https://cancer.sanger.ac.uk/cosmic). These mutations are supposed to drive disease [21–24]. We hypothesized that leukemic cells derived from these patients show downregulation of IFN responses.

**Fig. 7:**
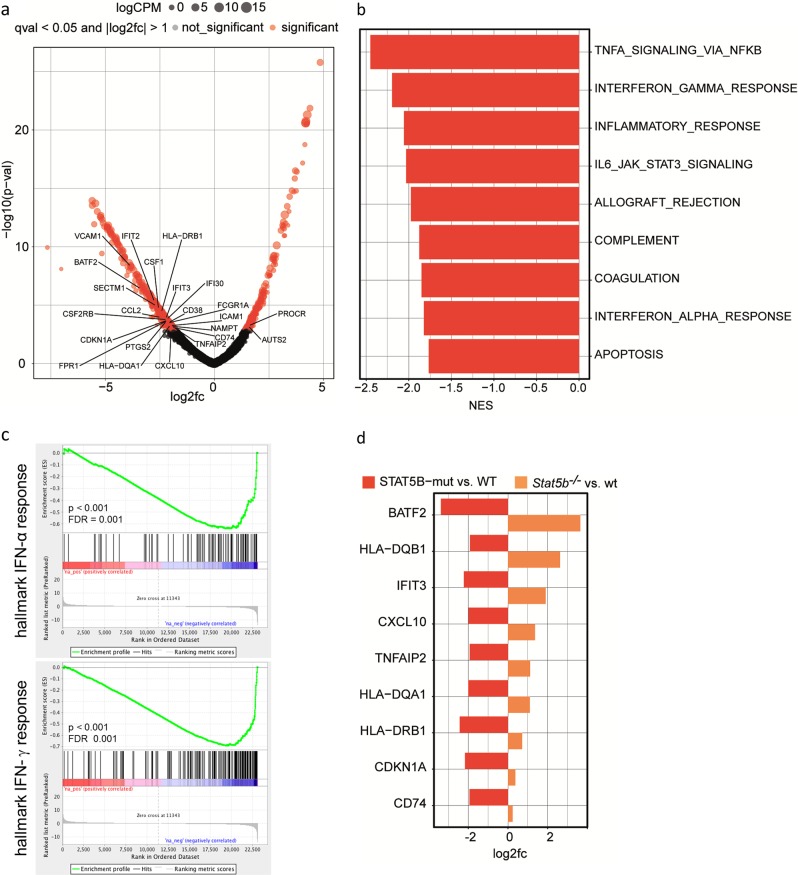
IFN responses are enhanced in human STAT5B mutant T cell large granular lymphocyte leukemia (T-LGLL). **a** RNA-seq from STAT5B mutant (*n* = 4) and wt (*n* = 13) T-LGLL patient samples. Significantly differentially (*q* value <0.05 and fold change >2) upregulated (*n* = 256) or downregulated (*n* = 376) genes were identified. Deregulated genes belonging to IFN-α and IFN-γ hallmark gene sets are indicated. Size of points corresponds to logCPM (counts per million) expression levels. **b** Significantly (NES <−1.75, FDR <0.25) negatively enriched hallmark gene sets obtained from GSEA of log 2 fold-change ranked gene lists from differential expression analysis of STAT5B mutant (vs. wt) T-LGLL cases. **c** Enrichment plots of the IFN-α and IFN-γ hallmark gene sets from GSEA. **d** Genes significantly (*p*-adjust <0.05) upregulated in *Stat5b*^*−/−*^ BCR/ABL^p185^ cell lines and significantly (*p*-adjust <0.05) downregulated in STAT5B mutant T-LGLL cases. IFN interferon, STATB signal transducers and activators of transcription 5B, RNA-seq RNA-sequencing, wt wild type, GSEA geneset enrichment analysis, NES normalized enrichment score, FDR false discovery rate

To test this, we performed RNA-seq of human STAT5B mutant T-LGLL samples using STAT5B-wt T-LGLL samples as controls. We identified 256 significantly upregulated and 376 significantly downregulated genes (Fig. 7a and Supplementary Table 3; *p*-adjust <0.05 and fold change >2). GSEA of this DEA revealed mainly negatively enriched gene sets, including IFN-α and IFN-γ responses (Fig. 7b, c). Only one gene set (HEME metabolism) was positively enriched (data not shown).

The opposite gene regulation pattern of human *STAT5B* mutant and murine *Stat5b*^*−/−*^ leukemias regarding IFN responses prompted us to compare the expression of a specific set of genes. We selected genes that were upregulated in the murine *Stat5b*^*−/−*^ samples and explored their expression in human *STAT5B* mutant leukemia. This approach identified nine IFN hallmark genes regulated in opposite directions: *BATF2*, *HLA-DQB1*, *IFIT3*, *CXCL10*, *TNFAIP2*, *HLA-DQA1*, *HLA-DRB1*, *CDKN1A*, and *CD74* (Fig. 7d).

## Discussion

Here, we investigated the role of STAT5 isoforms in BCR/ABL^+^ leukemia, a disease that ceases upon combined loss of STAT5A and STAT5B [9]. The use of *Stat5a*^*−/−*^- and *Stat5b*^*−/−*^-knockout mice allowed investigating individual contributions of STAT5B and STAT5A to the development of disease.

Our study defines STAT5B as the critical isoform for the transformation of BCR/ABL^+^ cells. Several lines of evidence support this conclusion; the enforced expression of STAT5B in BCR/ABL^+^ cells enhances cell proliferation, while knockdown decreases colony formation in vitro. In murine models, the lack of STAT5B prolongs tumor formation in vivo.

The privileged role of STAT5B for BCR/ABL-induced leukemogenesis is documented in reduced colony formation in the absence of STAT5B. The absence of STAT5A exerts minor effects. Similarly, the establishment of immortalized cell lines is severely hindered in the absence of STAT5B and demands enhanced levels of the BCR/ABL oncoprotein, whereas the absence of STAT5A is of minor impact. Once established, *Stat5b*^*−/−*^ cells maintain a reduced proliferative capacity and induce leukemia significantly later. Confirmation stems from experiments where we applied a leukemia model using retroviral infection in new-born mice: *Stat5b*^*−/−*^ mice succumbed significantly later to leukemia. In contrast, *Stat5a*^*−/−*^ cells—while showing a reduced but still existent capacity to transform in vitro—showed superimposable kinetics of disease onset as wt cells in both in vivo models.

The model of new-born infection adds another layer of complexity as STAT5B is absent in the entire organism including immunological tumor surveillance. In BCR/ABL-induced leukemia NK cells represent the primarily responsive cellular compartment which survey the tumor cells [31, 35, 36]. The fact that leukemia appears delayed in *Stat5b*^*−/−*^ mice came rather surprising as *Stat5b*^*−/−*^ NK cells are less functional—which would rather promote leukemia formation ([37, 38] and own observations). In contrast to our expectations that an impaired NK cell-mediated tumor surveillance accelerates leukemogenesis, STAT5B-deficient animals disease significantly later. This indicates a pronounced defect of *Stat5b*^*−/−*^ mice in tumorigenesis, which cannot be outweighed by a decreased NK cell-mediated tumor surveillance. It also indicates that any therapeutic strategy blocking STAT5B will maintain beneficial effects despite inhibiting NK cell functions.

STAT5B is critical at two distinct steps of transformation; during the initial switch from a normal to a transformed cell as well as during the establishment and progression of leukemia in vivo. The role of STAT5B in the initial phase of transformation is reflected by the reduced capability to form colonies. Blocking of IFN signaling restored the capability of *Stat5b*^*−/−*^ BM cells to transform and form colonies. The transcriptional programs driven by IFN-α and IFN-γ are significantly increased in *Stat5b*^*−/−*^ cells and only to a lesser extent in *Stat5a*^*−/−*^ BCR/ABL^+^ cells. An involvement of STAT5 in IFN signaling in other cellular systems has already been suggested [39–42]. Our study now poses the IFN–STAT5 axis in the core of B-lymphoid tumorigenesis.

Despite undetectable IFN levels via enzyme-linked immunosorbent assay (data not shown), we observed a clear-cut phenotype upon blocking signaling via interfering antibodies. Treatments with anti-IFNAR1 or anti-IFN-γ restored the ability of *Stat5b*^*−/−*^ BM cells to transform—pointing at the involvement of tonic IFN signaling. Tonic IFN signaling is characterized by a constant but low-level production of IFNs [43, 44]. Besides, during the initial transformation, cells encounter oncogenic stress and need to overcome the p53 response. In the latter, STAT5 was already predicted to have a critical role [45–47].

Even upon overcoming this initial hurdle and when the leukemic cell lines are established, STAT5B-deficient cells still display a significant disadvantage as fatal disease occurs with a delay in vivo. Here, the established role of STAT5B as cell cycle regulator comes into play; STAT5B controls cell cycle progression and its loss interferes with proliferation and motility of tumor cells [20, 27, 48–50]. We currently do not know how significant the STAT5B-mediated suppressive effect on IFN signaling is during this advanced stage of transformation. The fact that IFNs have been used as therapeutics for decades to treat patients suffering from BCR/ABL^+^ leukemia suggests a key role for IFN in this disease [44, 51–53]. Any therapeutic strategy that interferes with STAT5B signaling might also enhance IFN production of the leukemic cells that may support a curative effect.

Our data may be of direct clinical relevance: current studies propose STAT5B as a key driver in human hematological tumors [20–24]. In that sense, the activation of STAT5B might be an asset during transformation and explain why gain-of-function mutations are found in hematological disorders at increased frequency. Our analysis of patient-derived samples with *STAT5B* mutations confirms this concept and extends it to the human hematopoietic system. Patients suffering from lymphoid malignancies with an activating *STAT5B* mutation display deregulated IFN signaling pathways. We found many genes being mirror-inverted when compared to the murine *Stat5b*-deficient leukemic cells.

Our results contribute to the knowledge of how STAT5B controls tumorigenesis in the hematopoietic lineage, a finding that was originally described in STAT5B gain-of-function mouse models [15–17].

We provide a novel edge to the role STAT5B in transformation, besides controlling cell cycle, apoptosis, and p53 responses. STAT5B is required to suppress IFN signaling, which is necessary for cells to fully transform. A better understanding of the complex role of STAT5B in leukemia will enable the development of precision medicine strategies to treat disease.

### Availability of data and materials

Due to constraints in the ethical permit, the raw sequencing data of patients is only available from the corresponding author upon reasonable request.

## Supplementary information

Supplementary appendix

Supplementary table 1

Supplementary table 2

Supplementary table 3
