# Supplementary material for: Twins with different personalities: STAT5B—but not STAT5A—has a key role in BCR/ABL-induced leukemia
Source: Leukemia. 2019 Jan 24;33(7):1583–97. doi: 10.1038/s41375-018-0369-5 (PMC6755975; doi:10.1038/s41375-018-0369-5)
Supplement: Supplementary file 1 — Supplementary appendix [file 41375_2018_369_MOESM1_ESM.pdf]

SUPPLEMENTARY APPENDIX

Supplement to: Kollmann, Grundschober, Maurer *et al.* Twins with different personalities: STAT5B – but not STAT5A – has a key role in BCR/ABL-induced leukemia

Content

INVESTIGATORS 2

METHODS 3

Cell culture and dose response curves 3

Overexpression and shRNA-mediated knockdown of *Stat5a* or *Stat5b* 3

qPCR analyses 5

Flow cytometry analysis, antibodies, and cell sorting 5

Immunoblotting and intracellular staining 6

REFERENCES 8

Supplementary Figure 1. Enforced expression of *Stat5a* or *Stat5b* in a murine v-Abl transformed cell line. 9

Supplementary Figure 2. Outgrowth and imatinib-sensitivity of BCR/ABLp185+ cell lines. 10

Supplementary Figure 3. Transplantation of BCR/ABL^{p185+} cell lines. 12

Supplementary Figure 4. RNA-seq analysis of BCR/ABL^{p185+} cell lines derived from *wt*, *Stat5a*$^{-/-}$ and *Stat5b*$^{-/-}$ mice. 13

Supplementary Figure 5. GSEA analysis: significantly enriched (NES > 1, FDR < 0.25) hallmark gene sets obtained by GSEA (*Stat5b*$^{-/-}$ vs. *Stat5a*$^{-/-}$ BCR/ABL^{p185+} cell lines). 14

Supplementary Figure 6. Enrichment plots of selected significantly enriched (FDR<0.25) hallmark genes of IFN-α- and IFN-g responses, MTORC signalling as well as MYC-targets. 15

Supplementary Figure 7. Changes in expression of hallmark IFN-g response genes which contribute to core enrichment in GSEA in comparisons of *Stat5a*$^{-/-}$ (vs. *wt*) and *Stat5b*$^{-/-}$ (vs. *wt*) BCR/ABL^{p185+} cell lines. 16

Supplementary Figure 8. Impaired cell cycle progression upon knockdown of STAT5B in K562 cells. 17

Supplementary Figure 9. Absolute colony numbers upon blockage of IFN-α or IFN-γ. 18

Supplementary Table 1. Up- and downregulated genes in BCR/ABL^{p185+} *Stat5a*$^{-/-}$ vs. *wt* cells 19

Supplementary Table 2. Up- and downregulated genes in BCR/ABL^{p185+} *Stat5b*$^{-/-}$ cells vs. *wt* cells 19

Supplementary Table 3. Up- and downregulated genes in human STAT5B-mutant T-LGLL vs. STAT5-wt T-LGLL samples. 19

INVESTIGATORS

Twins with different personalities:

STAT5B – but not STAT5A – has a key role in BCR/ABL-induced leukemia

Sebastian Kollmann[a*], Eva Grundschober[a*], Barbara Maurer[a*], Wolfgang Warsch[a], Reinhard Grausenburger[a], Leo Edlinger[a], Jani Huuhtanen[b], Sabine Lagger[c], Lothar Hennighausen[d], Peter Valent[e,f], Thomas Decker[g], Birgit Strobl[h], Mathias Mueller[h], Satu Mustjoki[i], Andrea Hoelbl-Kovacic[a#], Veronika Sexl[a#]

[a] Institute of Pharmacology and Toxicology, University of Veterinary Medicine Vienna, A-1210 Vienna, Austria
[b] Hematology Research Unit Helsinki, University of Helsinki, FI-00290 Helsinki, Finland
[c] Unit of Laboratory Animal Pathology, University of Veterinary Medicine Vienna, A-1210 Vienna, Austria.
[d] Laboratory of Genetics and Physiology, National Institute of Diabetes and Digestive and Kidney Diseases, National Institutes of Health, Bethesda, Maryland, USA
[e] Department of Internal Medicine I, Division of Hematology and Hemostaseology, Comprehensive Cancer Center, Medical University of Vienna, A-1090 Vienna, Austria
[f] Ludwig Boltzmann Cluster Oncology, Medical University of Vienna, A-1090 Vienna, Austria
[g] Max F. Perutz Laboratories (MFPL), University of Vienna, A-1030 Vienna, Austria
[h] Institute of Animal Breeding and Genetics; Department for Biomedical Sciences, University of Veterinary Medicine Vienna, A-1210 Vienna, Austria
[i] Helsinki University Hospital Comprehensive Cancer Center, University of Helsinki, FI-00290 Helsinki, Finland
*These authors contributed equally to this work.
[#] Shared last authorship.

METHODS

Cell culture and dose response curves

The murine Ab-MuLV^{+} (v-Abl), BCR/ABL^{p185+} and human K562 cell lines were established and cultured in RPMI medium (Sigma-Aldrich, St. Louis, MO, USA) supplemented with 10% fetal calf serum (FCS), 50 µM 2-mercaptoethanol, 100 U/ml penicillin, and 100 µg/ml streptomycin (PAA) as described[1]. 5x10^{5} BCR/ABL^{p185+} cell lines were incubated with imatinib as described[2]. Dose response curves (DRCs) were performed as described[3]. Briefly, after 24 h of incubation, CellTiter-Glo (Promega, Fitchburg, WI, USA) was added and luminescence measured with an Envision plate reader (Perkin Elmer, Waltham, MA, USA)[4].

Overexpression and shRNA-mediated knockdown of *Stat5a* or *Stat5b*

Overexpression: *wt* murine v-ABL^{+} cells were infected with a pMSCV-IRES-GFP based construct encoding *Stat5a* or *Stat5b* as described[5]. Infections with the empty vector served as control.

Knockdown studies: Retroviral shRNA LENC vectors (encoding shStat5a, shStat5b or shRenilla (shRen; control (Ctr)) and eGFP) were prepared according to the manufacturer´s instructions (Thermo Scientific, Waltham, MA, USA).

The murine target sequences of the vectors are as follows:

Insert/gene/ Info	Sequence (5' > 3')
shStat5a	TGCTGTTGACAGTGAGCGATCCTGTTTGAGTCTCAGTTCATAGTGAA GCCACAGATGTATGAACTGAGACTCAAACAGGACTGCCTACTGCCTC GGA
shStat5b #1	TGCTGTTGACAGTGAGCGCAGGCGTCTCCTTGAAGGACAATAGTGAA GCCACAGATGTATTGTCCTTCAAGGAGACGCCTTTGCCTACTGCCTC GGA
shStat5b #2	TGCTGTTGACAGTGAGCGATCAAGTGGAGATGTTTAACAATAGTGAA GCCACAGATGTATTGTTAAACATCTCCACTTGAGTGCCTACTGCCTCG GA

and shRen as described[6].

Generation of retroviral supernatant and infections of cells were performed as described[7].

Lentiviral shRNA pRRL vectors (encoding shSTAT5A, shSTAT5B or shRenilla (shRen; control (Ctr)) were prepared according to the manufacturer´s instructions[8].

The human shRNA sequences are as follows:

Insert/gene/Info	Guide	97mer
shSTAT5A #1	TTGATCTGAAG GTGCTTCTGGG	TGCTGTTGACAGTGAGCGACCAGAAGCAC CTTCAGATCAATAGTGAAGCCACAGATGT ATTGATCTGAAGGTGCTTCTGGGTGCCTA CTGCCTCGGA
shSTAT5A #2	TGTTACTACAG GAAGGAGCGGA	TGCTGTTGACAGTGAGCGCCCGCTCCTTC CTGTAGTAACATAGTGAAGCCACAGATGT ATGTTACTACAGGAAGGAGCGGATGCCTA CTGCCTCGGA
shSTAT5B #1	TTTAACACTTCC ATCACACCGT	TGCTGTTGACAGTGAGCGCCGGTGTGATG GAAGTGTTAAATAGTGAAGCCACAGATGT ATTTAACACTTCCATCACACCGTTGCCTAC TGCCTCGGA
shSTAT5B #2	TTGACTTGAAA AACCAGCTCAT	TGCTGTTGACAGTGAGCGCTGAGCTGGTT TTTCAAGTCAATAGTGAAGCCACAGATGT ATTGACTTGAAAAACCAGCTCATTGCCTA CTGCCTCGGA
shSTAT5B #3	TATACTTCATCT TTTGGCCGAT	TGCTGTTGACAGTGAGCGCTCGGCCAAAA GATGAAGTATATAGTGAAGCCACAGATGT ATATACTTCATCTTTTGGCCGATTGCCTAC TGCCTCGGA

and shRen as described[6].

Lentiviral particles were produced as previously described with some modifications[9]. Described shRNA constructs were co-transfected with packaging and envelope plasmids psPAX2 and pMD2.G in human embryonic kidney cells (HEK293FT) using Lipofectamine 2000 (Life Technologies) in a 10cm tissue culture dish. 72 hours post transfection, viral supernatant was harvested, concentrated with the PEG virus precipitation kit (BioVision, Inc), aliquoted and stored at -80°C.

$5x10^5$ K562 cells were transduced with 30µl of viral supernatant in the presence of 10µg/ml hexadimethrine bromide (Sigma) for 72 hours.

qPCR analyses

RNA was isolated using the RNeasy MiniKit (Qiagen, Hilden, Germany). RNA was transcribed with the iSCRIPT cDNA synthesis kit (Bio-Rad). Quantitative real-time PCR was performed on a CFX96 Real-Time System C1000Touch Thermal Cycler (Bio-Rad) with SsoAdvanced universal SYBR GreenSupermix (Bio-Rad). Following primers were used:

Human:

gene name	forward (5'>3')	reverse (5'>3')
STAT5B	GAACGCATCTGCAGATGCCG	TCAAGGACTGAGTCAGGG
BATF2	AGACCCCAAGGAGCAACA	CTTTTTCCAGAGACTCGTGCT
CXCL10	CCAGAATCGAAGGCCATCAA	CATTTCCTTGCTAACTGCTTTCAG
STAT1	ATGCGGTTGAACCCTACACG	GTTCCATTGGCTCTGGTGC
GAPDH	TCTCCTCTGACTTCAACAGCG	ACCACCCTGTTGCTGTAGCC

Target gene expression was normalized to expression of Glyceraldehyde 3-phosphate dehydrogenase (GAPDH).

Murine:

gene name	forward (5'>3')	reverse (5'>3')
Mx-1	GACTACCACTGAGATGACCCAGC	ATTTCCTCCCCAAATGTTTTCA
Ifn-γ	AAGTGGCATAGATGTGGAAG	GAATGCATCCTTTTTCGCCT
Ifn-α	CATCTGCTGCTTGGGATGGAT	TTCCTGGGTCAGAGGAGGTTC
Rplp0	GCTTTCTGGAGGGTGTCC	GCTTCAGCTTTGGCAGGG

Target gene expression was normalized to expression of Ribosomal Protein Lateral Stalk Subunit P0 (*RPLP0*).

Flow cytometry analysis, antibodies, and cell sorting

Single-cell suspensions were analyzed by a BD FACS Canto II flow cytometer equipped with 488, 633, and 405 nm lasers using FACS Diva software (Becton-Dickinson) as described before[4]. For surface marker staining, suspensions were pre-incubated with αCD16/CD32 (FCγIII/II receptor; BD) antibodies to prevent nonspecific Fc-receptor-mediated binding. Subsequently, cells were stained with monoclonal antibodies conjugated

with fluorescent markers and analysed by a FACSCantoII flow cytometer using FACSDiva software (Becton-Dickinson). The following antibodiy was used: Anti-mouse CD19 eFluor450 (Clone: eBio1D3, eBioscience).

For propidium iodide (PI) and apoptosis staining 1x10^6 cells were analysed. PI staining was performed with PI (50 µg/ml) and incubated at 37°C for 30 minutes[4]. Analysis of apoptotic cells was conducted according to the manufacturer´s instructions (Annexin V Apoptosis Detection Kit eFluor® 450, 88-8006, eBioscience). For cell cycle analysis, 1 x 10^6 cells were stained with PI (50 µg/ml) in a hypotonic lysis solution (0.1% sodium citrate, 0.1% triton X-100, 100 µg/ml RNAse) and incubated at 37°C for 30 minutes.

eGFP$^+$ cells were high-purity FACS sorted on a FACS Aria III equipped with a 488 nm laser at 4 °C using FACSDiva software version 6.1.2 (Becton-Dickinson).

Immunoblotting and intracellular staining

Whole cell lysates were harvested as described[4]. Proteins were separated on a 7% SDS polyacrylamide gel and transferred to nitrocellulose membranes. The following antibodies were used for immunoblotting: c-Abl (Cell Signalling, Danvers, MA, USA, 2862S), pSTAT1 (S727) (Cell Signalling, 9177S), pSTAT1 (Y701) (Cell Signalling, 7649S), pSTAT2 (Y689) (Merck Millipore, Billerica, MA, USA, 07-224), STAT1 (Cell Signalling, 9172L), STAT2 (Merck Millipore, 07-140). α-Tubulin (DM1A, Santa Cruz) or ß-actin (AC-15, Santa Cruz) served as loading control. Anti-mouse and anti-rabbit HRP conjugated secondary antibodies were used (Cell Signalling, 7074S and 7076S). Chemiluminescent visualisation of the bands was performed with a ChemiDocTM Touch Imaging System (Bio-Rad, Hercules, CA, USA) after incubation of the membranes with Clarity Western ECL reagent (Bio-Rad).

For intracellular staining 3 x 10^6 cells were fixed by 2% paraformaldehyde (Aldrich)/PBS at 37°C for 10 minutes. All washing steps were performed for 10 minutes with 10 ml PBS/2% FCS/0.2% Tween-20 per sample. Cells were washed twice and permeabilized with 99% ice-

cold methanol for 30 minutes at 4°C. Cells were washed twice and incubated with αCD16/CD32 and pSTAT5-PE (Y694, 12-9010-41, ebioscience) at RT for one hour. Cells were washed two times before analysing via flow cytometry.

REFERENCES

1. Kovacic B, Stoiber D, Moriggl R, et al. STAT1 acts as a tumor promoter for leukemia development. *Cancer Cell*. 2006;10(1):77–87.
2. Warsch W, Kollmann K, Eckelhart E, et al. High STAT5 levels mediate imatinib resistance and indicate disease progression in chronic myeloid leukemia. *Blood*. 2011;117(12):3409–20.
3. Grundschober E, Hoelbl-Kovacic A, Bhagwat N, et al. Acceleration of Bcr-Abl + leukemia induced by deletion of JAK2. *Leukemia*. 2014;28(9):.
4. Berger A, Hoelbl-Kovacic A, Bourgeais J, et al. PAK-dependent STAT5 serine phosphorylation is required for BCR-ABL-induced leukemogenesis. *Leukemia*. 2014;28(3):629–41.
5. Warsch W, Grundschober G, Berger A, et al. STAT5 triggers BCR-ABL1 mutation by mediating ROS production in chronic myeloid leukaemia. *Oncotarget*. 2012;3(12):.
6. Fellmann C, Hoffmann T, Sridhar V, et al. An Optimized microRNA Backbone for Effective Single-Copy RNAi. *Cell Rep.* 2013;5(6):1704–1713.
7. Edlinger L, Berger-Becvar A, Menzl I, et al. Expansion of BCR/ABL1$^+$ cells requires PAK2 but not PAK1. *Br. J. Haematol.* 2017;179(2):.
8. Warlich E, Kuehle J, Cantz T, et al. Lentiviral Vector Design and Imaging Approaches to Visualize the Early Stages of Cellular Reprogramming. 2011;
9. Lagger S, Meunier D, Mikula M, et al. Crucial function of histone deacetylase 1 for differentiation of teratomas in mice and humans. *EMBO J.* 2010;29(23):3992–4007.

Supplementary Figure 1

a

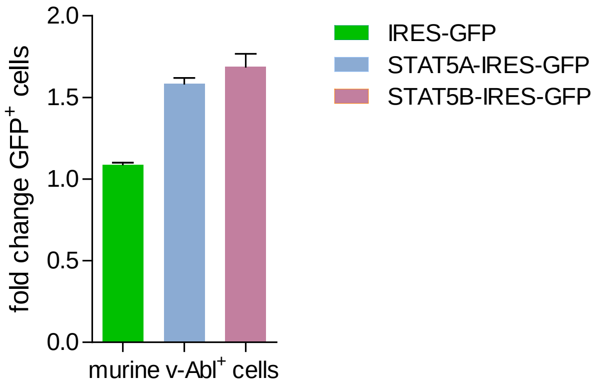

b

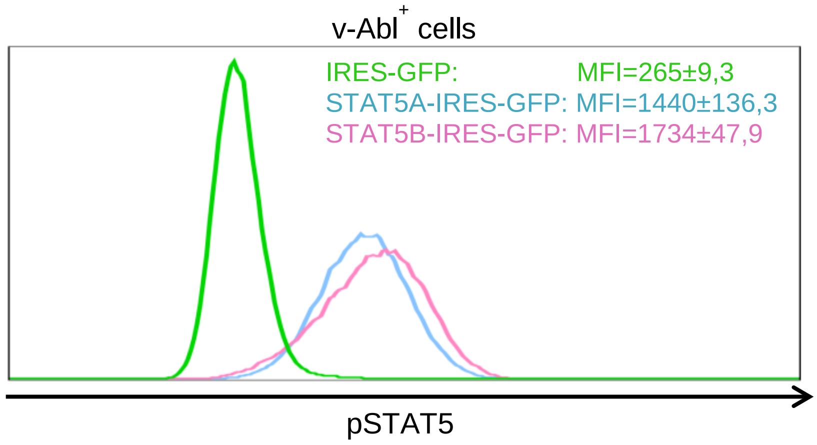

c

Infection Rates of total BM cells	IRES-GFP	STAT5A-IRES-GFP	STAT5B-IRES-GFP
%GFP+ cells	4.1 ±0.1	4.9 ±0.1	4.9 ±0.3

Supplementary Figure 1. Enforced expression of *Stat5a* or *Stat5b* in a murine v-Abl transformed cell line.

(a) Fold change of GFP+ cells after 18 days of competitive growth with their uninfected counterparts are plotted (n=2 cell lines/genotype). Error bars represent mean ±SEM. Levels of significance were calculated using Kruskal-Wallis-test followed by Dunn's test.

(b) pSTAT5 (Y694) levels of v-Abl+ cells with induced expression of STAT5A-IRES-GFP, STAT5B-IRES-GFP or GFP (empty vector). **(c)** Infection rates at day 1 of total BM with STAT5A-IRES-GFP, STAT5B-IRES-GFP or GFP corresponding to **Figure 1c**.

Supplementary Figure 2

a

genotype	established cell lines	time to stable cell line	GFP-MFI (BCR-ABL)
wt	12/12 (100%)	~ 6 weeks	6262 ± 684
Stat5a$^{-/-}$	10/17 (59%)	~ 6 weeks	8574 ± 3674
Stat5b$^{-/-}$	4/14 (29%)	~12 weeks	10719 ± 2519

b

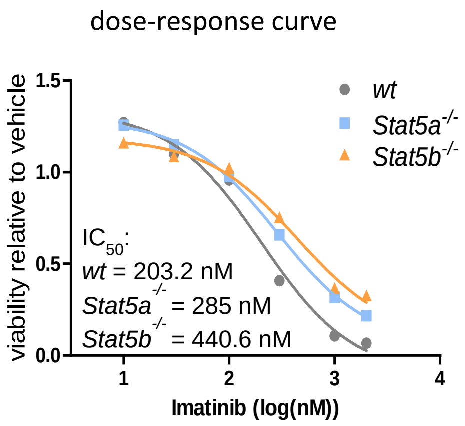

Supplementary Figure 2. Outgrowth and imatinib-sensitivity of BCR/ABLp185+ cell lines.

(a) *wt*, *Stat5a*$^{-/-}$ or *Stat5b*$^{-/-}$ BM cells were transduced with a retrovirus encoding BCR/ABL^{p185}. Each cell line derived from an individual mouse. MFI: mean fluorescence intensity. **(b)** Dose-response curves of *wt*, *Stat5a*$^{-/-}$ or *Stat5b*$^{-/-}$ BCR/ABL^{p185+} cells treated with raising concentrations of imatinib. Levels derived from treatment with DMSO served as control and were set to 1 (n=3 cell lines/genotype).

Supplementary Figure 3

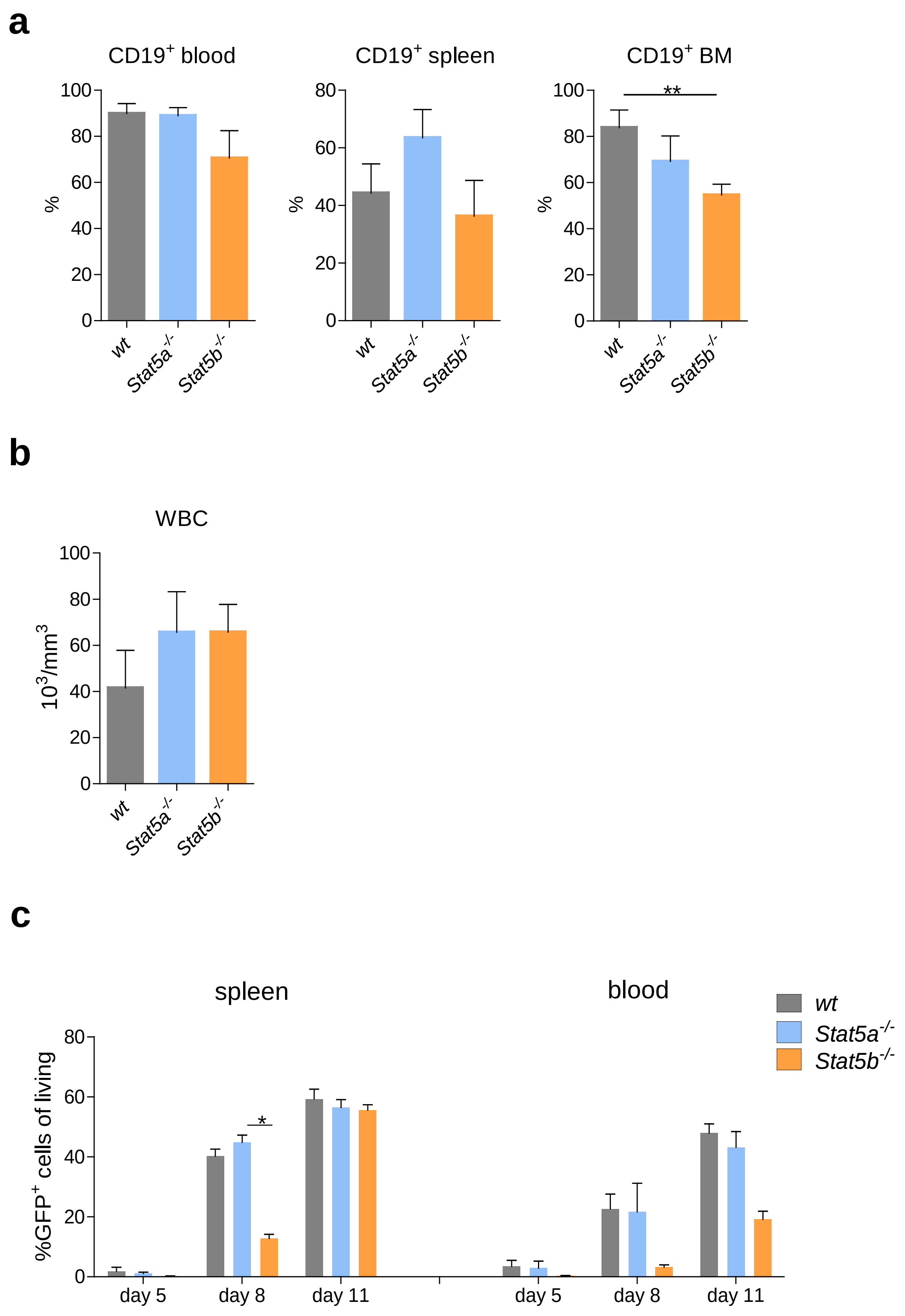

Supplementary Figure 3. Transplantation of BCR/ABL^{p185+} cell lines.

(a) Percentages of CD19^{+} cells in spleen, bone marrow and blood (n≥5 per genotype). Error bars represent mean ± SEM. Levels of significance were calculated using one-way ANOVA. **(b)** White blood cell (WBC) counts of mice which have reveived *wt*, *Stat5a*$^{-/-}$ and *Stat5b*$^{-/-}$ BCR/ABL^{p185+} cells (n≥5 per genotype). Error bars represent mean ± SEM. Levels of significance were calculated using one-way ANOVA. **(c)** i.v. injection of *wt*, *Stat5a*$^{-/-}$ or *Stat5b*$^{-/-}$ BCR/ABL^{p185+} cells into NSG mice. Three mice per genotype were sacrificed on day 5, day 8 and day 11 upon injection of BCR/ABL^{p185+} cells. Quantitative analysis via FACS for GFP^{+} BCR/ABL^{p185+} cells in spleen and blood (n=3 per genotype and day). Error bars represent mean ± SEM. Levels of significance were calculated using Kruskal-Wallis-test followed by Dunn’s test.

Supplementary Figure 4

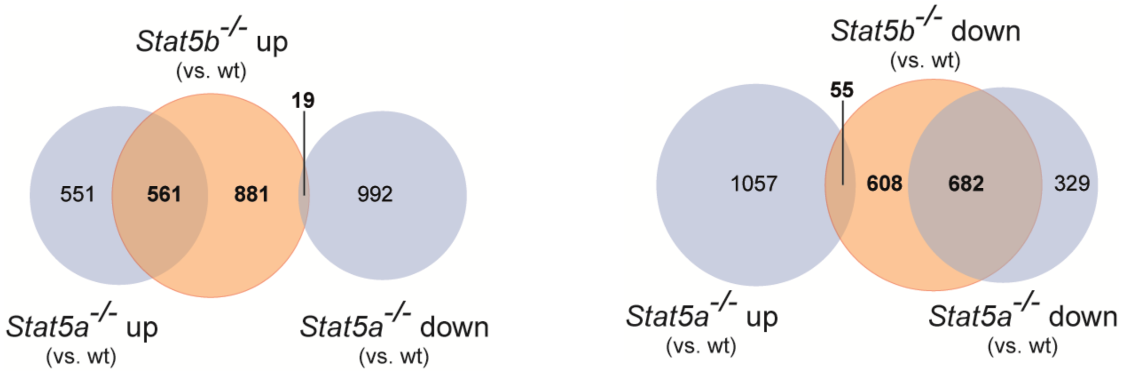

Supplementary Figure 4. RNA-seq analysis of BCR/ABL^{p185+} cell lines derived from *wt*, *Stat5a*$^{-/-}$ and *Stat5b*$^{-/-}$ mice.

Venn diagram of significant differentially expressed (fold change > 2, padjust < 0.1) up- and down-regulated genes of *Stat5b*$^{-/-}$ (vs. *wt*, orange) and *Stat5a*$^{-/-}$ (vs. *wt*, blue) BCR/ABL^{p185+} cell lines.

Supplementary Figure 5

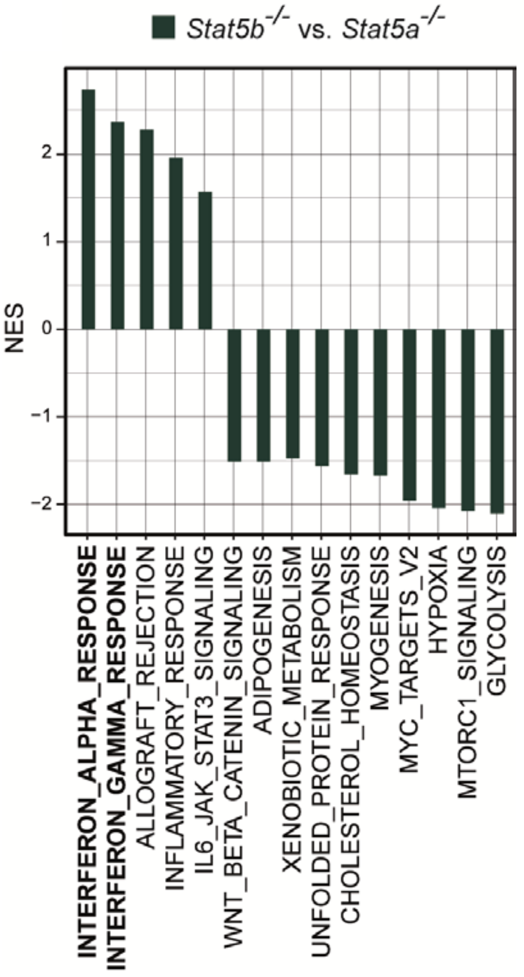

Supplementary Figure 5. GSEA analysis: significantly enriched (NES > 1, FDR < 0.25) hallmark gene sets obtained by GSEA (*Stat5b*$^{-/-}$ vs. *Stat5a*$^{-/-}$ BCR/ABL^{p185+} cell lines).

Supplementary Figure 6

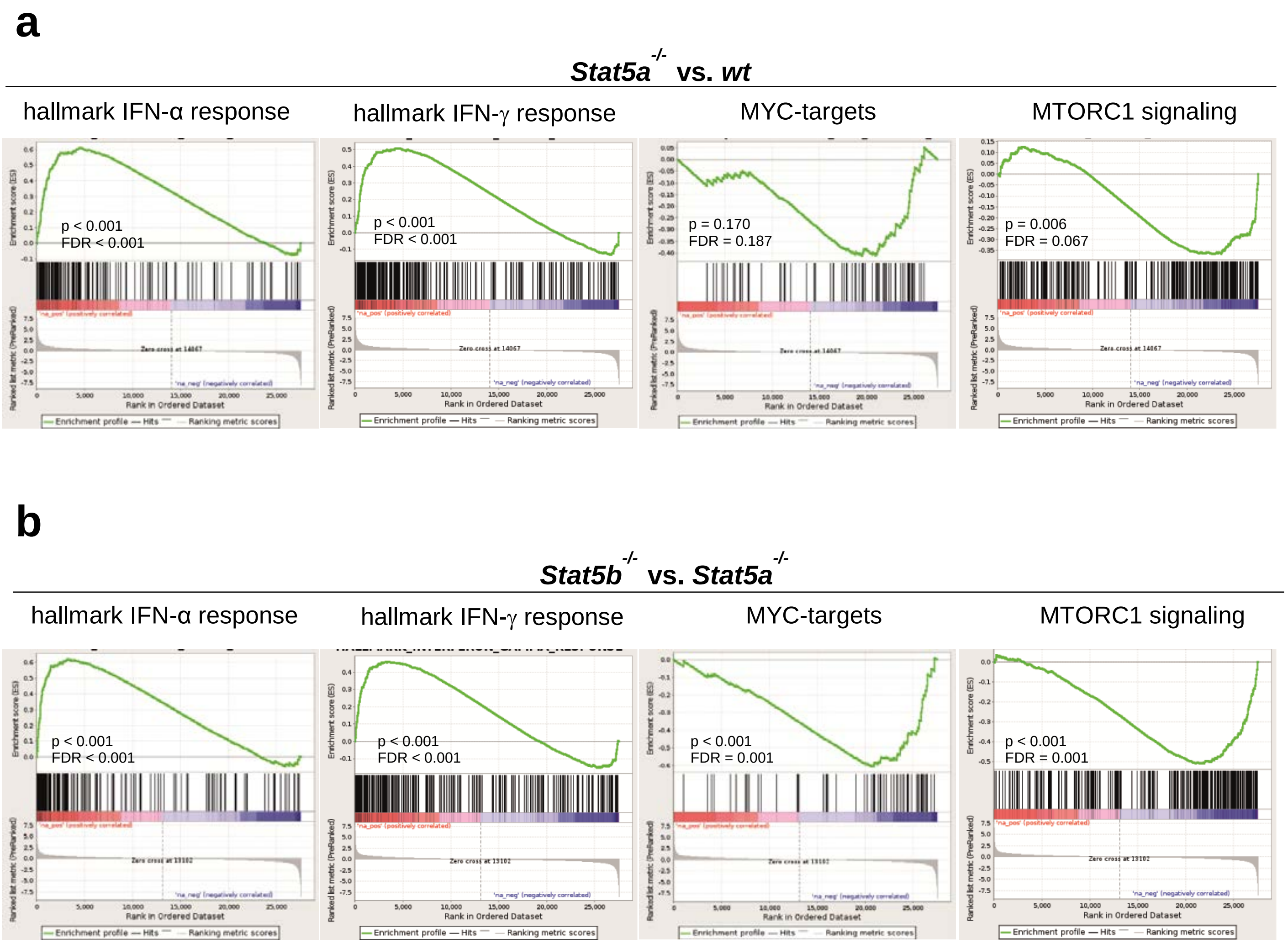

Supplementary Figure 6. Enrichment plots of selected significantly enriched (FDR<0.25) hallmark genes of IFN-α- and IFN-γ responses, MTORC signalling as well as MYC-targets.

Data were obtained by GSEA of log2 fold change ranked, differentially expressed genes between **(a)** *Stat5a*$^{-/-}$ vs. *wt* and **(b)** *Stat5b*$^{-/-}$ vs. *Stat5a*$^{-/-}$ BCR/ABL^{p185+} cell lines.

Supplementary Figure 7

hallmark IFN-γ response genes

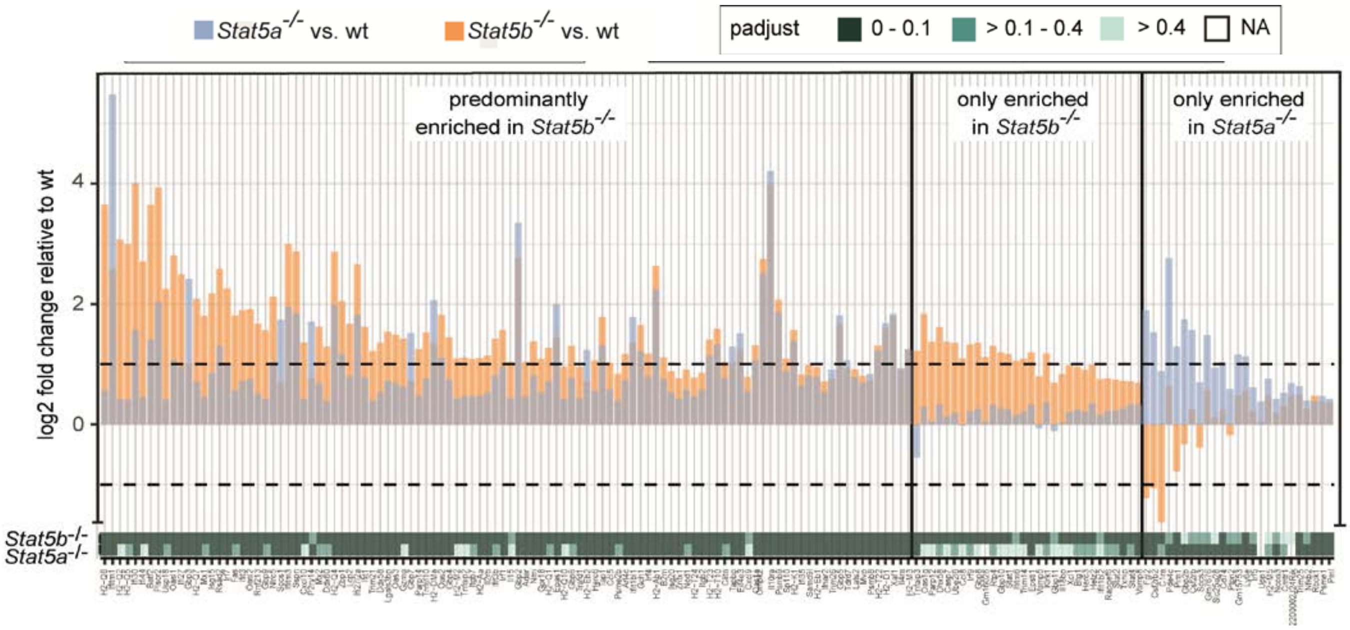

Supplementary Figure 7. Changes in expression of hallmark IFN-γ response genes which contribute to core enrichment in GSEA in comparisons of *Stat5a*$^{-/-}$ (vs. *wt*) and *Stat5b*$^{-/-}$ (vs. *wt*) BCR/ABL^{p185+} cell lines.

The log2 fold change and the adjusted p-value of the differential expression analysis are shown. IFN-γ response genes are grouped into genes which contribute to GSEA core enrichment in both *Stat5a*$^{-/-}$ (vs. *wt*) and *Stat5b*$^{-/-}$ (vs. *wt*) BCR/ABL^{p185+} cell lines, only in *Stat5b*$^{-/-}$ (vs. *wt*), and only in *Stat5a*$^{-/-}$ (vs. *wt*) differential expression analysis.

Supplementary Figure 8

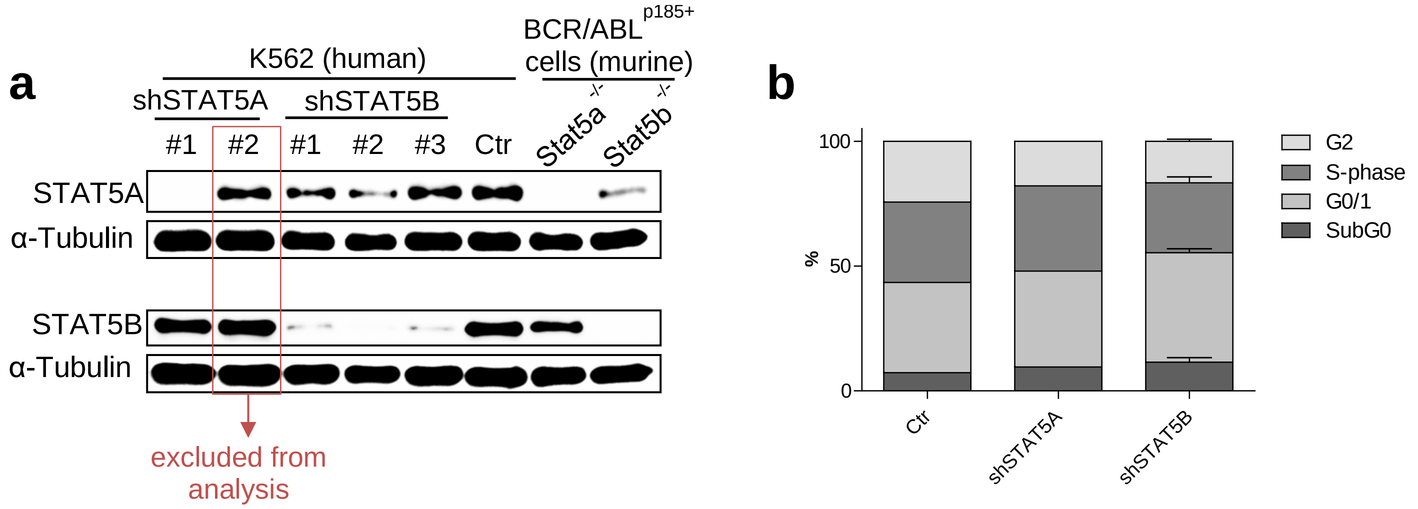

Supplementary Figure 8. Impaired cell cycle progression upon knockdown of STAT5B in K562 cells.

(a) Knockdown efficiencies of different shRNAs against STAT5A or STAT5B on K562 cells via immunoblot analysis. **(b)** Cell-cycle profiles analyzed by PI-staining of K562 cells illustrated in (a) (n=3 for shSTAT5B). Error bars represent mean ± SEM.

Supplementary Figure 9

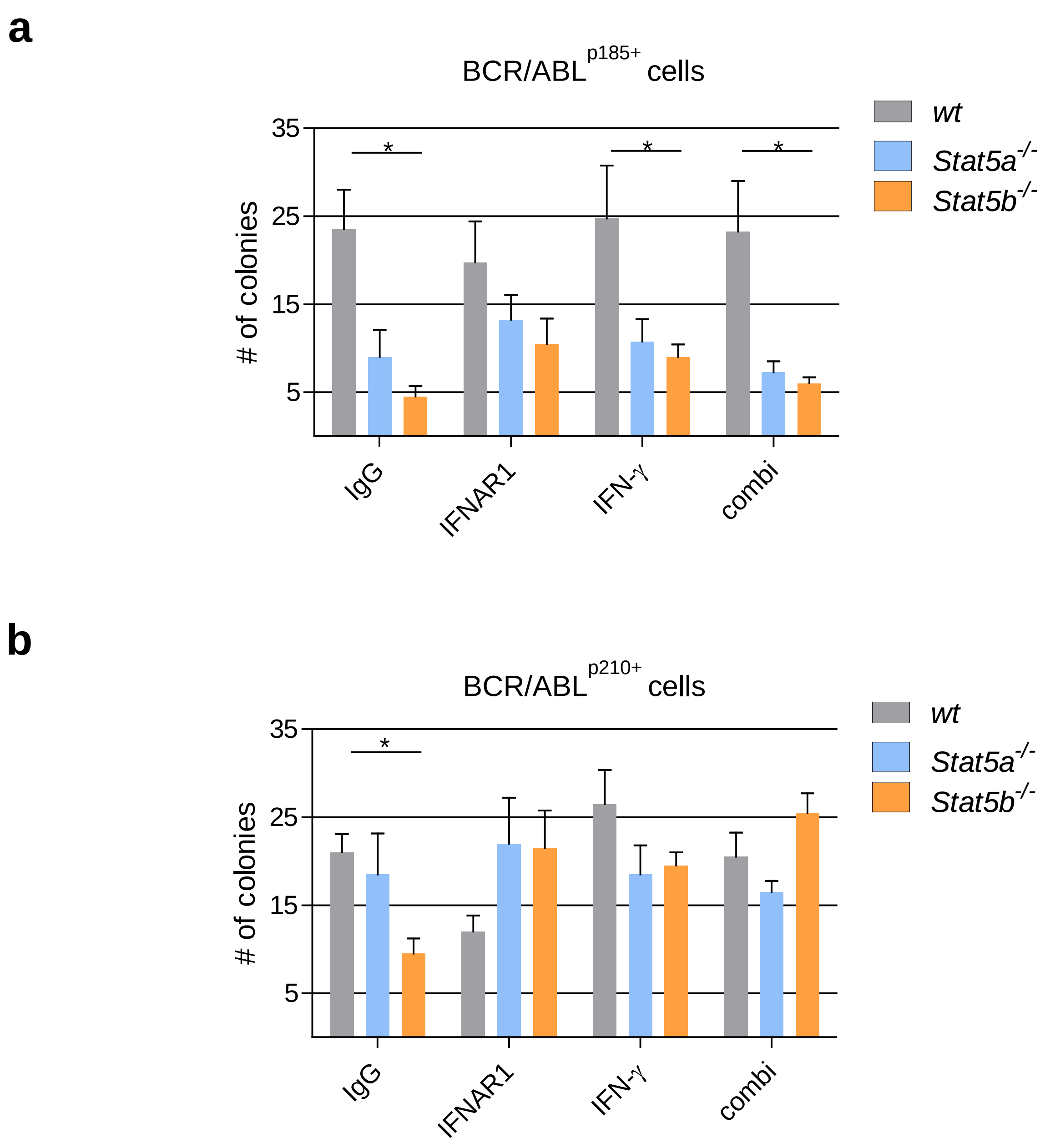

Supplementary Figure 9. Absolute colony numbers upon blockage of IFN-α or IFN-γ. BM cells of *wt*, *Stat5a*$^{-/-}$ or *Stat5b*$^{-/-}$ mice were infected with a retrovirus encoding **(a)** BCR/ABL^{p185} or **(b)** BCR/ABL^{p210} and plated in growth-factor free methylcellulose. In addition 10µg/ml antibodies (Ab) against IgG blocking IFNAR1 or IFN-γ or combination of both (in total 10µg/ml) were added (n=4 per genotype). Error bars represent means ±SEM. Levels of significance were calculated using Kruskal-Wallis-test followed by Dunn’s test.

Supplementary Table 1. Up- and downregulated genes in BCR/ABL^{p185+} *Stat5a*$^{-/-}$ vs. *wt* cells

available in a separate xls.file

Supplementary Table 2. Up- and downregulated genes in BCR/ABL^{p185+} *Stat5b*$^{-/-}$ cells vs. *wt* cells

available in a separate xls.file

Supplementary Table 3. Up- and downregulated genes in human STAT5B-mutant T-LGLL vs. STAT5-wt T-LGLL samples.

available in a separate xls.file
